# 

**Published:** 1892-04-23

**Authors:** 


PRICE TWOPENCE.

No. 291, Vol. XII.—April 23rd, 1892.

[Registered at the General Post Office as a Newspaper for transmission in the United Kingdom and Abroad.]

WITH EXTRA NURSING SUPPLEMENT
Per Annum, 8s. 6d., post free.
Monthly Parts, 6d.; post free, 8d.
Ditto per Annum, 8s., post free.

### THE HOSPITAL

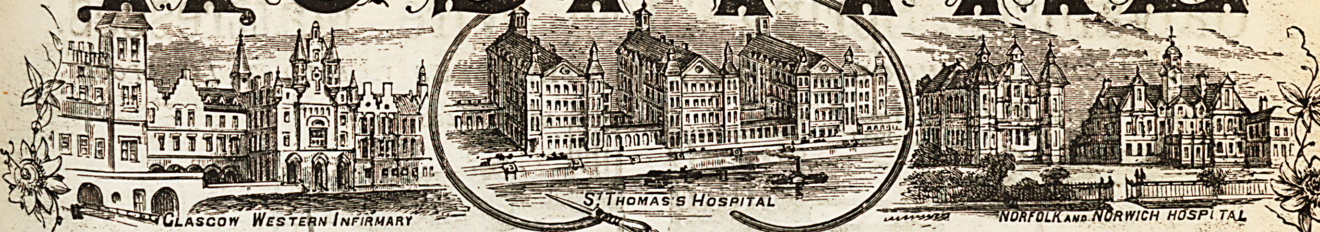

WITH EXTRA NURSING SUPPLEMENT.

No. 291. Vol. XII.] SATURDAY, APRIL 23rd, 1892. [TWOPENCE.

